# Further Remarks on the Use of Ether in Obstetrical Practice

**Published:** 1848-03

**Authors:** Jonathan Clark


*Further Remarks on the Use of Ether in Obstetrical Practice. Report of Cases in continuation of those published in the Number of the Examiner for October*, 1847. By Jonathan Clark, M.D.

Case 7th.—At 2 o'clock, P. M., on the 16th of October, I saw Mrs. ——, of Roxborough, aged 20, in labour with her first child.

Her pains had come on early in the day, and had increased gradually in frequency and force until I saw her, when they were severe and frequent. There was a most distressing nausea, with vomiting occasionally of acid matter. On examining her *per vaginam*, the *os uteri* appeared to be very little dilated. The head was presenting, but high up in the pelvis.

An antacid mixture was administered, with a view of correcting the acid state of the stomach; when, as there was little prospect of a rapid progress, I left her for a time. Returning at 4 o'clock, there was still little progress; nausea and vomiting, about the same as before. I now took 16 oz. of blood from the arm. This had a marked effect on the state of the os uteri, which dilated much more rapidly, enabling me to determine the position of the head to be with the vertex to the left acetabulum.

As she was anxious to inhale the ether, having seen its effects in a like case of one of her neighbours, I administered it. This was at half past 4 o'clock; in three minutes she was fully under its influence. Her pulse, which had been 85 when she commenced, rose to 90 beats in the minute, and continued at this rate for a short time, and then fell to 79, and remained at that rate.

The expulsive efforts were not so frequent, when she was in a complete state of lethargy, as when she appeared to be partly conscious, and showed indications of some suffering. After she had been under the influence of the ether for three hours, I resolved to deliver with the forceps. The length of time elapsing since the labour had commenced, and the prospect of its continuance for some hours longer, were, I thought, sufficient reasons for a resort to this alternative. I should mention, that from the first inhalation, the nausea and vomiting had entirely ceased. Having seen it stated somewhere, that it was safest, or most prudent, to apply the instruments while the patient was *not* under the influence of the ether, thus having the feeling of the patient as a guide to prevent injury during their application, I withheld the sponge till she became apparently conscious of what was occurring near her, answered questions, and vehemently demanded more ether.

With some trouble, owing to the contracted or rather undilated state of the os externum, and to the position of the head, the vertex being far to the left, I succeeded in adjusting the blades with less than the usual amount of suffering. Renewing the inhalation till a complete lethargy was induced, and then during the expulsive efforts of the uterus, making guarded efforts to extract the head, I, after the third pain, was successful, having renewed the inhalation twice during the efforts. There was no difficulty in determining when a pain was present, even when the patient was in a profound lethargy, more or less motion being communicated to the forceps by the contraction of the uterus. The child, a female of average size, was vigorous, and did well. The placenta came away in a short time, without the knowledge of the patient. In six minutes, being eight minutes after the child was delivered, she regained her consciousness.

She had not any recollection of the birth of the child, nor yet of the application of the forceps. This was a little remarkable, as I had discontinued the use of the ether for a time while applying them, and had thought from her manner, that she was not under its influence. No untoward symptoms showed themselves until the fifth day, when she was seized in the morning with a violent chill, followed by fever and profuse perspiration. A repetition of the same symptoms occurred on the following day. This difficulty yielded to mercurials and quinine in a couple of days; since which she has convalesced pleasantly, and is now well.

Case 8th. At 7 o'clock, A.M., October 28th, I was requested to see Mrs. ——, aged 22, of Lower Merion, in labour with her first child. She had been all night in labour. The pains were not of a severe character, although they prevented her from obtaining any rest. An examination *per vaginam* showed the *os uteri* to be dilated to the size of a sixpence, and quite rigid. The membranes had been ruptured spontaneously some time previously. As there was little progress making, I left her and returned at noon.

An examination then showed the head to be presenting, the os uteri dilated to the extent of three inches, and dilatable, the vertex to the left acetabulum. Quite severe pains were recurring at intervals of two and a half to three minutes. They were more annoying than effective in forwarding the labour, seeming to lack in a remarkable degree expulsive power.

As her patience was well nigh exhausted, I proposed the ether, a means of relief which was acceded to. Her pulse was 112 when she commenced inhaling; in two minutes it fell to 80. This was at half past 1 o'clock. The pains were not quite as frequent as before inhaling the ether. She lay with her eyes closed, apparently free from suffering except when the ether evaporated from the sponge; she would then, during a contraction of the uterus, begin to make an effort to say something, squeezing the sponge and holding it very *tightly* to her face. In the next pain, however, she would remove the sponge, and call for more: "quick, quick, give me more."

I was fearful that my supply of ether would not hold out, having but a limited quantity, and the slow progress of the case indicating that she would have to be kept under its influence for a considerable time. An attempt to economise by putting less on the sponge, soon occasioned her to demand more, when I told her that she must not use it fast or it would not hold out; she replied very sharply, "that is your look out."

The length of time she had been in labour, together with the slow progress the case was making, and the certainty of seeing her suffer severely when the ether was gone, induced me to resort to the forceps. I made an effort to withhold the ether previous to their application, but she would not listen to any such arrangement, nor the reasons for it, but completely overwhelmed me with solicitations for more whenever I attempted to explain. I yielded to her wishes, and applied the instruments while she was in a complete lethargy, taking great care to use no force whatever. With unusual facility I succeeded in adapting the instruments to the head, and by gentle traction at proper intervals delivered the child, a large female, which has done well. The placenta came away in a short time, but not until she had recovered her sense of pain, and wished much to take some more ether on account of it. She had no knowledge whatever of the use of the instrument or the birth of the child. She recovered pleasantly without an unfavourable symptom, and is now quite well.

Case 9th.—Oct. 30th, at 7 o'clock, A. M., I saw Mrs. ——, aged 20, of Manayunk, in labour with her first child. She had been suffering pain at intervals since the day before, and had slept none during the night. At the time I saw her the pains were quite regular, recurring at intervals of three minutes. After waiting a short time, I made an examination, which showed the os uteri to be slightly dilated, and dilateable, the membranes entire, and the head presenting with the vertex to the left acetabulum. She was anxious to take the ether, having some weeks previously made arrangements for that purpose, by procuring a supply of it, and a sponge, fearing I might neglect to bring them when called to attend her. At a quarter to nine o'clock she commenced inhaling the vapour, and in two minutes dropped the sponge, being in a complete lethargy.

This did not last, however, more than three minutes, when a pain came on, which partially roused her, when she again grasped the sponge and renewed the inhalation. The labour progressed slowly but regularly. When the os uteri was sufficiently dilated I ruptured the membranes during a pain.

I observed that, although my patient was in a perfecly insensible state, the force with which the liquor amni was ejected was as great as I had witnessed in any case heretofore, showing that the contractile power of the uterus was not impaired. When she had been seven hours under the influence of the ether, and in actual labour twelve or thirteen, there was a prospect of its continuing some hours longer, owing to the obstruction offered by the contracted state of the os externum: I resolved to deliver with the forceps. I did not withhold the ether, but applied them while she was completely under its influence, taking great care to use no unusual force. In ten minutes from the time when the instruments were locked, she was delivered of a female child of large size, without any injury to the perineum. An assistant was directed by her to apply the sponge to her nostrils whenever her sensibility was returning, and she inhaled thus, unobserved by me, more than I wished, during the time occupied in applying the forceps, and delivering her. In eight minutes she aroused from her lethargy, but was not aware of the birth of the child nor of the use of the instruments. The placenta came away in a short time. There was considerable flooding, but not sufficient to cause alarm. What was rather unusual, it being a first case, was the occurrence of sharp after-pains, which yielded to an anodyne. The child was vigorous and healthy. On the eighth day, owing to an unpardonable error in diet, there was a severe chill, followed by a train of alarming symptoms, great depression, profuse perspiration, vomiting and diarrhœa. This difficulty yielded to appropriate treatment in a few days, and she is now doing well.

Case 10th.—At 8 o'clock, A. M., Nov. 2d, I was requested to see Mrs. ——, of Blockley, aged 24, in labour with her third child. The pains had come on during the previous evening, and had continued all night. They were regular and tolerably severe when I saw her. An examination *per vaginam* showed dilatation of the os uteri to the extent of nearly three inches. The membranes were entire, and the head presented with the vertex to the right *sacro-iliac* junction. The *os uteri* was quite dilatable, and the head pretty well down in the pelvis. Therefore, after waiting a short time, I ruptured the membranes, and succeeded in bringing the vertex under the right acetabulum. The patient was of an irritable temperament, and bore her pains with very little fortitude.

The case being a favourable one, I proposed using the ether. Some of her acquaintances who were present injudiciously alarmed her by asking whether I had heard of certain cases in which it had had a bad effect, which occasioned her to decline using it for a time; but the pains becoming more severe, on being assured that it would not injure her, she consented to inhale it. In three minutes she was fully under its influence.

The pains were not so frequent as they were before she used it, but quite as efficient. When the effects of an inhalation were passing off, she would refuse to inhale again until she had almost regained her reasoning powers. There seemed to be an impression on her mind, even under etherization, that it would injure her; and this idea continued to influence her conduct until she had almost or quite regained her power of reasoning, when another train of ideas would take its place, and she would again very willingly inhale. This conduct was, I thought, owing to the remarks of her attendants; one of whom seemed to be much annoyed to see the poor woman getting through her labour without feeling each pain as acutely as she herself had done, as the mother of several children.

In thirty minutes from the time she first used the ether, she gave birth to a large male child. She was conscious at the time of its birth, but seemed to suffer very little, and expressed herself much pleased with the relief afforded by the inhalation. There was more flooding than I had observed in any case in which I had used the ether. The placenta came away promptly. When she had recovered from the effects of the ether, she complained very much of after-pains, which yielded to moderate doses of morph. sulph. She is convalescing pleasantly.

CASE 11th.—December 12th, at 4 o'clock, P. M., I saw Mrs. ——, of Lower Merion, age 30, in labour with her third child. She had been suffering some hours.

At the time I saw her the pains were very severe, recurring at intervals of two minutes and a half, and of three-fourths of a minute duration. The pulse was 68 in the minute. An examination, *per vaginam*, shewed that no progress was made in the labour. With considerable effort I failed to reach the *os uteri.* This was owing to distortion of the pelvis, which had very much protracted her former labours, in both of which I had attended her. Although she had been suffering severe and regular pains for at least three hours, the membranes having been ruptured spontaneously, the head appeared not to have entered the superior strait, and I was unable to determine the presentation, or even reach the *os uteri*, without introducing my hand into the vagina. This I thought unnecessary. As there was great anterior protrusion of the abdomen in the hypogastric region, she was placed on her back, and, as that position was not disagreeable to her, she retained it during the labour. The remembrance of what she had suffered during her former labours, caused her to be very desponding in this. She complained so much, as to induce me to administer the ether sooner than I should otherwise have done. From what I had observed in other cases, I had a suspicion that it might modify somewhat the expulsive efforts of the uterus, and I was aware from former experience in her case, that all its powers would be required to overcome the obstruction offered by the contracted superior strait.

With some reluctance she inhaled the ether, commencing at half past five o'clock. It had a stimulating effect, increasing the pulse to 79. Although it was administered freely, it did not produce lethargy, neither did it prevent her from complaining as much as she did before she took it. She talked more during the intervals between the pains, though in a discursive and incoherent manner, than she did previous to the inhalation. The pains were not modified in frequency or duration, and were, if any thing, more violent. The relief which I expected to see follow the inhalation of the ether, not having been obtained, I concluded to discontinue its use. In a few minutes she began to complain more violently than ever, and begged most earnestly for more of the ether, saying that "it afforded her so much relief." This surprised me. It was, however, apparent on a readministration of it, that her complaints did not evince such suffering and acute anguish, as when she was not under its influence.

A re-examination, *per vaginam*, showed that the labour was progressing, a part of the child could now be felt; but I failed to determine the presentation. It was with difficulty that the anterior margin of the *os uteri* could be felt.

The presenting part descended very gradually into the pelvis, until I was enabled to determine that it was the head, but so distorted and tumefied as to prevent me from discerning the presentation. This eventually proved to be such as to bring the vertex under the arch of the pubis. As the labour progressed, she became very sensible of the relief obtained from the ether, and frequently requested to have the sponge moistened.

She continued to talk incessantly when the pain was off, and when she was most fully under the influence of the ether, she appeared to be in a very happy mood, saying how happy she was, what a blessing it was to have such relief, &c. Among other expressions, she said she had become simple, quite childish, but had never expected to live so long as to become childish.

At a quarter of ten o'clock, the head escaped from the superior strait, and in a few minutes, the child, a boy of medium size, was delivered. Its head was very much elongated and distorted, compressing the brain so much, as to prevent it from showing signs of vitality for a time, but by the use of appropriate means it revived, and eventually did well. The placenta came away in a few minutes, and there was considerable after-pain.

No case has come under my notice, in which more gratitude was expressed for the relief afforded by the ether, and in none has the relief been less apparent. The power of appreciating, and recollecting pain and suffering, appeared to be destroyed by the inhalation, while the ability to evince its presence was still retained. There was, during the greater part of the labour, both before and after the etherization, a repetition of chills, and previous to the inhalation, nausea with vomiting, but none afterwards. The patient convalesced more rapidly, having retained more strength, than on any previous occasion.

Case 12th.—On the 23d of December, at 2 o'clock, A. M., I saw Mrs. ——, aged 31, of Lower Merion, in labour with her third child. She was in the eighth month of her pregnancy, and had been exerting herself in an unusual degree for some days before. An examination showed that but little progress had been made in the labour. I left her for some hours; and when I returned, as there was little prospect of a speedy delivery, I again left her and did not return till 4 o'clock, P. M., when there was a highly favourable change.

The head was presenting, with the vertex to the left of the acetabulum; the membranes were entire, and almost protruding without the *os externum*. As the pains were severe, and she wished it, the ether was administered.

After inhaling for five minutes she was only partially under its influence, as she would not inhale properly. It however afforded her great relief. The pains did not diminish in force or frequency. In half an hour, she was delivered of a female child, which was very feeble, being evidently premature. It moaned for near an hour, and expired. The placenta came away in a few minutes, and there was considerable after-pain, which yielded to a moderate dose of morph. sulph. Convalescence was rapid, and unattended by any unfavourable symptoms. In this case, the patient seemed evidently relieved by the inhalation, at the same time she was aware of the birth of the child, and appeared perfectly conscious. The other children had been premature; this one had lived longer than either of the others.

Case 13th.—At ten o'clock in the evening of the 2d of January, I saw Mrs. ——, aged 30, of Lower Merion, in labour with her third child. She had been seized with pains at 7 o'clock, P. M. At the time I saw her, they were of one minute duration, with an interval of two minutes. The pulse was one hundred in a minute. An examination, *per vaginam*, showed the head to be presenting, and still in the superior strait. The os uteri was dilated to the size of a dollar, and dilatable. The vertex was to the left acetabulum.

At twenty minutes past one o'clock, A. M., when the pains had become very severe, and the labour had made considerable progress, the ether was administered. In five minutes she was but partially under its influence, owing to a reluctance, or perhaps an inability to inhale the vapour, as it occasioned some coughing, and she was afraid it would strangle her; owing to this cause she did not obtain its full effects for some time. Her pulse was not affected in the least, as far as I could perceive. The paroxysms of pain were shortened for a time, being reduced to forty-five seconds, and the intervals between the pains lengthened to three minutes; this state of things, however, was but temporary, for in a little while the pain became almost incessant. Having become aware of the relief afforded by the vapour, she would ask frequently to have the sponge wet with the ether, and would inhale with great earnestness. At ten minutes after two, she was delivered of a female child of small size, but very sprightly. It has done well. The placenta came away in six minutes. The after-pains were very severe, so much so, as to induce me to resort to the ether to relieve them. A few inhalations had a decided effect in modifying them, and relieving her suffering. They recurred again, however, on withholding the vapour. As I could not make it convenient to stay, and did not think proper to trust the patient or her friends with the remedy, I gave a moderate dose of morph. sulph., with directions to repeat it if necessary, and took my leave.

This patient was perfectly aware of what occurred during the time she was inhaling the vapour, still she found great relief from its use, and expressed herself as much pleased with its effects. Her convalescence was very satisfactory.

Case 14th.—On the seventh of January, at 4 o'clock, P. M., I was requested to see Mrs. ——, of Lower Merion, aged 18, in labour with her first child. She had slept none the night previous, owing to lingering pains which annoyed her very much. When I saw her, the pains were tolerably severe, occurring at intervals of five minutes. An examination showed that very little progress had been made in the labour, the *os uteri* not being perceptibly dilated. I left her and returned at 8 o'clock, when there was a highly favourable change apparent. The *os uteri* was well dilated, and the head was presenting, with the vertex to the right acetabulum, and well down in the pelvis. The pains were now recurring, at intervals of one and a half minutes, and were of one minute duration. As she was suffering severely, I proposed using the ether, when her friends informed me that I had been called on with special reference to its use. It was accordingly administered, and in three minutes a complete lethargy was induced. It had the effect to diminish the frequency of the pains somewhat, and also to lessen their duration.

This effect was most marked, when the lethargy was most complete, but I observed that a sufficient degree of insensibility could be maintained, to prevent suffering, without interfering with the progress of the labour.

In a short time the pains became very active and efficient, the patient being comparatively quiet, and appearing to suffer but little. This was the more remarkable as it was her first labour, and the head was so large as to distend the perineum exceedingly. At a quarter past ten o'clock, she gave birth to a very large female child, which was quite vigorous; being larger than usual, I had it weighed; its weight was nine pounds. The mother had no knowledge of the birth of the child, but regained her consciousness before the placenta came away, which was in eight minutes. She convalesced without an unpleasant symptom.

Case 15th.—On the 9th of January, at half past 11 o'clock, P. M., I saw Mrs. ——, of Blockley, aged 25, in labour with her third child. Her pains had increased gradually since 8 o'clock, at which time they had commenced, till the time of my seeing her, when they were quite severe, recurring at intervals of two minutes. An examination showed the *os uteri* to be well dilated, the head presenting, with the vertex to the left acetabulum, and the membranes entire. The head was still in the superior strait.

After rupturing the membranes, as she was suffering intensely, the pains having increased in force and frequency, the ether was administered.

She commenced inhaling at five minutes of 12 o'clock, when the pains were recurring at intervals of 70 seconds, and were of 35 seconds duration, varying however somewhat.

In two minutes she was under its influence sufficiently to render her unconscious, having no recollection of any thing, till after her delivery. The frequency of the pains was sensibly diminished, and they were not so regular.

When she became fond of inhaling, which she soon did, and induced a complete lethargy, one interval of four minutes occurred between the pains. She appeared to be in a loving mood, kissing very often, her mother-in-law, with whom she lived, and embracing her, wishing to be caressed in return. She would not permit her to cross the room without calling her to come and kiss her, often saying, "my dear mother, I do love you."

The pains were very efficient, and at half past one o'clock, she gave birth to a female child of average size. For fifteen minutes after its birth, she did not seem to be conscious of what had occurred, but on hearing the child cry she asked what it was. She frequently said, "oh, what a queer place I was at, mother, why did not you go with me? It was such a queer place." When I asked her how she felt, she looked at me with a mixture of doubt and astonishment in her countenance, and exclaimed, "oh doctor I thought I saw you there, now I know how it is," seeming to recollect herself. The placenta came away within five minutes. There were no unpleasant symptoms; on the contrary, she said, she had more strength and felt better than she had after either of her other labours. It is usual for ladies to say, that the last labour was harder than any previous one. Her head was clear and free from pain, or any unpleasant sensation, and she convalesced rapidly.

Case 16th.—January 13th, at 10 o'clock, P. M., I saw Mrs. ——, of Lower Merion, age 26, in labour with her third child. She had been in labour several hours, but for the last half hour the pains had been quite severe, and frequent.

An examination showed the os uteri to be dilated, to the extent of two inches, the membranes entire, and the head occupying the superior strait. The pulse was 82 to the minute. After rupturing the membranes, the ether was administered, and in three minutes she was under its influence, so as to remark, "I am going to sleep." When she arrived at this state she would take away the sponge, and cease to inhale, thus failing to obtain as much relief as I could have wished.

The pains were not modified in force or frequency, nor was the pulse affected. The labour progressed rapidly, and at twenty-five minutes of 11 o'clock, she was delivered of a male child of average size, which has done well, being quite vigorous.

The patient several times said she was going to sleep, but did not. On the contrary, she made more outcry, I thought, than if she had had not taken the ether. She once observed that she felt as if she had been drinking. When asked if she was aware of the birth of the child, she said she knew all about it, she had retained her consciousness throughout, but that the ether was a great relief to her. The placenta came away in a few minutes, no unfavourable symptoms occurred, and the mother and child are both doing well.

The foregoing cases exemplify some of the properties of etherization, heretofore noticed by others, and some which seem not to have been recorded. In general, any morbid state was rectified by the etherous influence, so that nausea and vomiting disappeared, convulsive tendencies were removed, and the pulse raised if previously depressed, and lessened in frequency, if antecedently too rapid.

In most cases, a perfect etherization destroyed the painfulness of labour, even when the patient retained consciousness. In other and rare instances, the sufferings were abated but not removed, even when the intellectual powers seemed lost for the time.

Generally, the duration and frequency of the labour-action were lessened, without any apparent retardation of the usual result of the labour; nor does there seem to have been any unusual proclivity to hemorrhagy in my cases.

On the whole, my trials of ether, as a torture-saving agent, have been satisfactory to me and my patients, and a sufficient number of cases have come under my notice, to justify me in the continuance of the use of a remedy which seems not only to save sorrow and suffering in common cases, but to be doubly useful when there are morbid complications. The extraordinary length of time during which delicate females can bear a strong etherization, seems to me to put at rest, if that has not been done by others, any reasonable doubt of its perfect safety, when used in the simplest manner, by respiring through a sponge.

